# External Root Resorption: Unique Cone Beam Computed Tomography Findings of a Rare Radiographic Entity

**DOI:** 10.7759/cureus.116293

**Published:** 2026-09-15

**Authors:** Karthik Rajaram Mohan, Ramachandra Reddy Gowda Venkatesha, Saramma Mathew Fenn

**Affiliations:** 1 Oral Medicine and Radiology, Vinayaka Mission's Sankarachariyar Dental College, Vinayaka Mission's Research Foundation (Deemed to be University), Salem, IND; 2 Oral Medicine and Radiology, Vinayaka Mission's Sankarachariyar Dental College, Vinayaka Mission's Research Foundation (Deemed to be university), Salem, IND

**Keywords:** cone beam computed tomography, endodontic treatment, external root resorption, mandibular premolar, radiographic interpretation

## Abstract

External root resorption occurs as a physiological or pathological process following pulpal necrosis. It ensues due to a traumatic insult to the pulp from underlying mechanical and chemical stimuli. The mechanical stimuli that favour external resorption include excessive orthodontic forces generated during orthodontic tooth movement, abnormal occlusal forces of mastication caused by traumatic occlusion, pressure effects caused by underlying impacted teeth, and odontogenic cysts and tumours. External resorption can also result from chemical stimuli such as bleaching, extruded filling materials, and intracanal medicaments. Such an interesting incidental finding of a case series of external root resorption occurring in a 54-year-old male around a horizontally impacted permanent right maxillary canine and in a 20-year-old male around an erupted right mandibular first premolar caused by impacted supernumerary teeth is discussed here.

## Introduction

External surface resorption (ESR) is a multifactorial process that involves exogenic and host-related factors [1]. The host-related factors include age, gender, ethnicity, hormonal disorders, chronic asthmatics, root shape, habits, malocclusion, and exogenous factors such as trauma, dental caries, pulpitis, orthodontic forces to the tooth, pulpotomy with calcium hydroxide, bleaching, and cracks on the tooth surface [1]. External resorption was classified as surface resorption, inflammatory resorption (apical and lateral), replacement resorption (progressive and transient), external pressure resorption, external invasive resorption, idiopathic resorption, and orthodontic resorption [2]. External root resorption occurs as an incidental finding during a radiographic examination, as it is often asymptomatic. The most prevalent resorption types identified by cone beam computed tomography were external (29.3%) and caused by pressure from impacted teeth (8.8%) [3]. Patel et al. classified external root resorption based on the height (1 = supracrestal, 2 = subcrestal in the coronal third of the root, 3 = subcrestal in the middle third of the root, 4 = subcrestal in the apical third of the root), three-dimensional circumferential extent of the lesion (A = ≤90°, B = ≤180°, C = ≤ 270°, D = >270°), accessibility, and proximity to the root canal (d = lesion confined to dentine, p = probable pulpal involvement) [4].

## Case presentation

Case 1

A 20-year-old male reported for a routine radiographic evaluation of his dentition. The cone beam computed tomography (CBCT) axial section revealed a bow-tie-like radiolucent appearance on the root of the right mandibular first premolar. The external root resorption is caused by pressure effects from lingually placed impacted supernumerary teeth. The CBCT sagittal section revealed an "M-shaped radiolucent appearance" extending from the external surface into the internal aspect of the root canal, involving the apical region of the root in the right mandibular first premolar and lingually placed supernumerary teeth (Figure 1A-1B).

**Figure 1:**
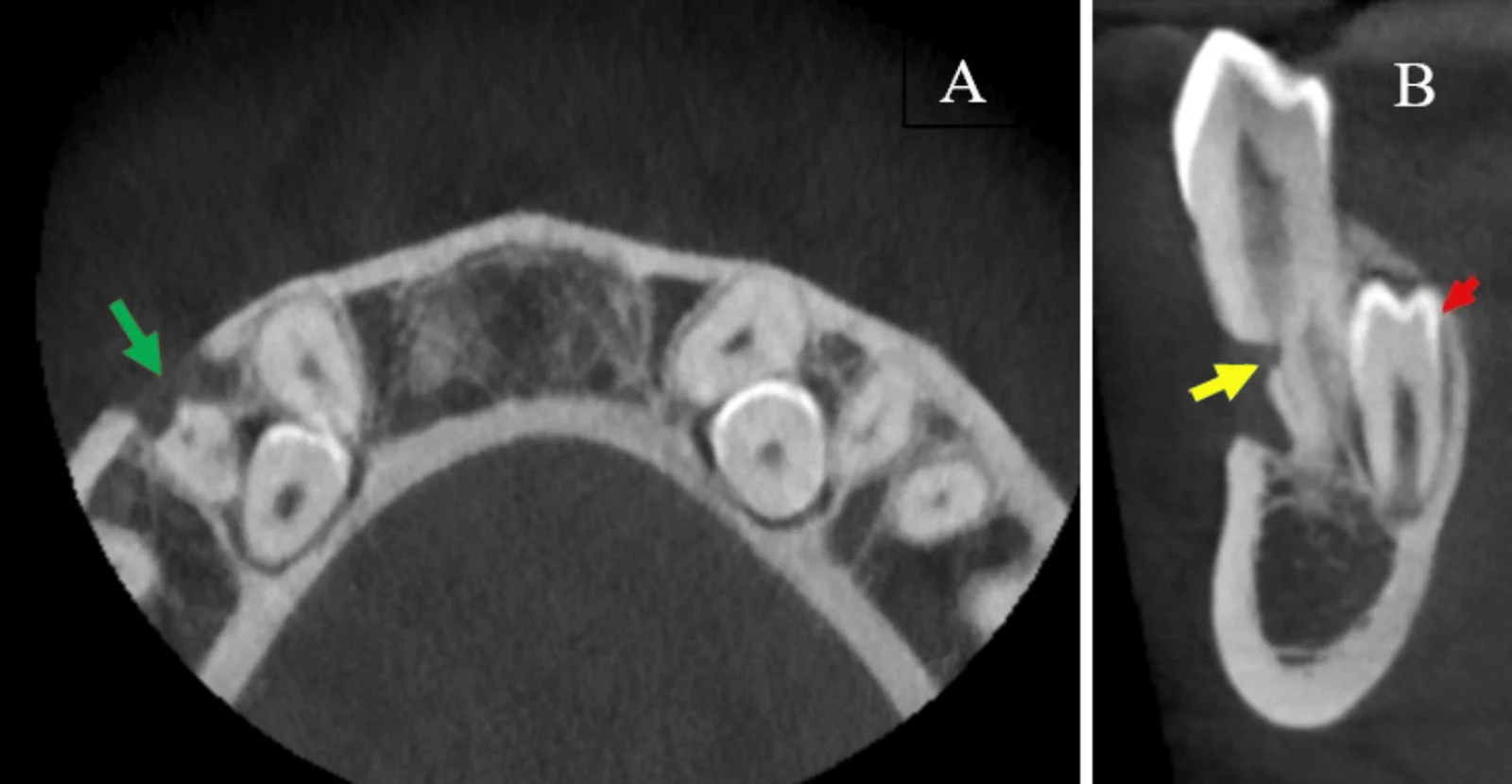
(A) CBCT axial section revealed "Bow-tie" like appearance of right mandibular first premolar (green arrow) and impacted supernumerary teeth. (B) CBCT sagittal section revealed a "M-shaped" radiolucency involving the root surface of right mandibular first premolar (yellow arrow) and lingually placed impacted supernumerary teeth (red arrow).

Case 2

A 54-year-old male was reported to our department for a radiographic evaluation of his missing tooth. His past dental history revealed he had undergone root canal treatment in the right maxillary lateral incisor and maxillary first premolar tooth regions two years ago. He also underwent a fixed prosthesis in a private clinic, which got accidentally dislodged and lost a month ago. Intraoral clinical examination revealed prepared abutment teeth in the right maxillary lateral incisor, right maxillary first premolar, and a small cusp-like tooth structure in the edentulous right maxillary canine region (Figure 2).

**Figure 2:**
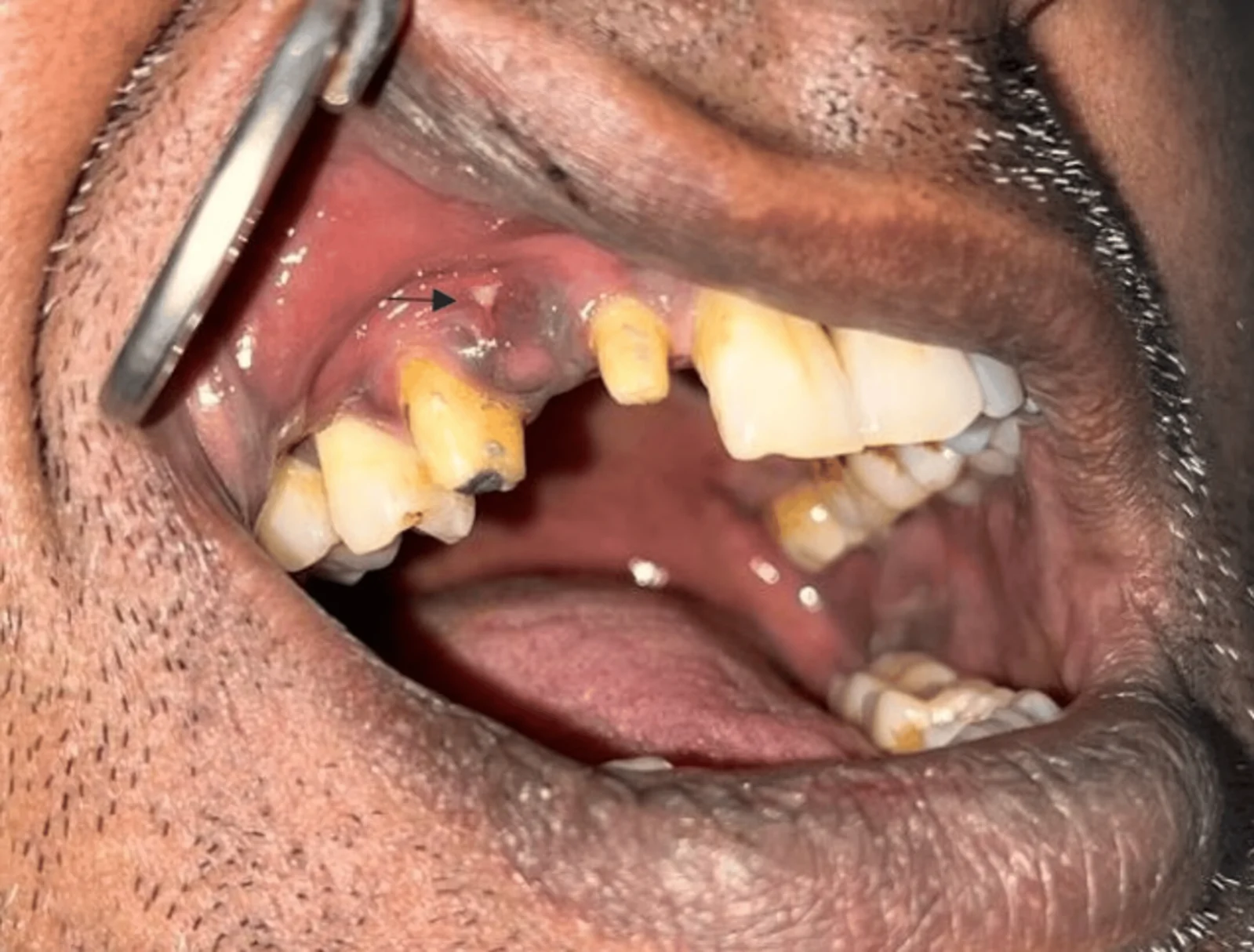
Intra-oral clinical examination revealed a small tooth-like cusp tip in the right maxillary anterior edentulous region.

The coronal section of the CBCT revealed an irregular radiolucent outline extending along the external aspect of the root of the right maxillary canine, involving underlying pulp and destruction of the palatal cortical plate. The CBCT sagittal section also revealed an irregular radiolucency near the CEJ (cementoenamel junction) of a horizontally impacted right maxillary canine. The axial section of CBCT showed an irregular radiolucent area extending from the root portion's external surface near the cementoenamel junction and involving the underlying pulp and an area of cortical destruction of about 5.3 mm × 3.8 mm. The three-dimensional (3D) reconstructed CBCT image revealed an impacted right maxillary canine and prepared teeth in the right maxillary lateral incisor and maxillary first premolar (Figure 3A-3D).

**Figure 3:**
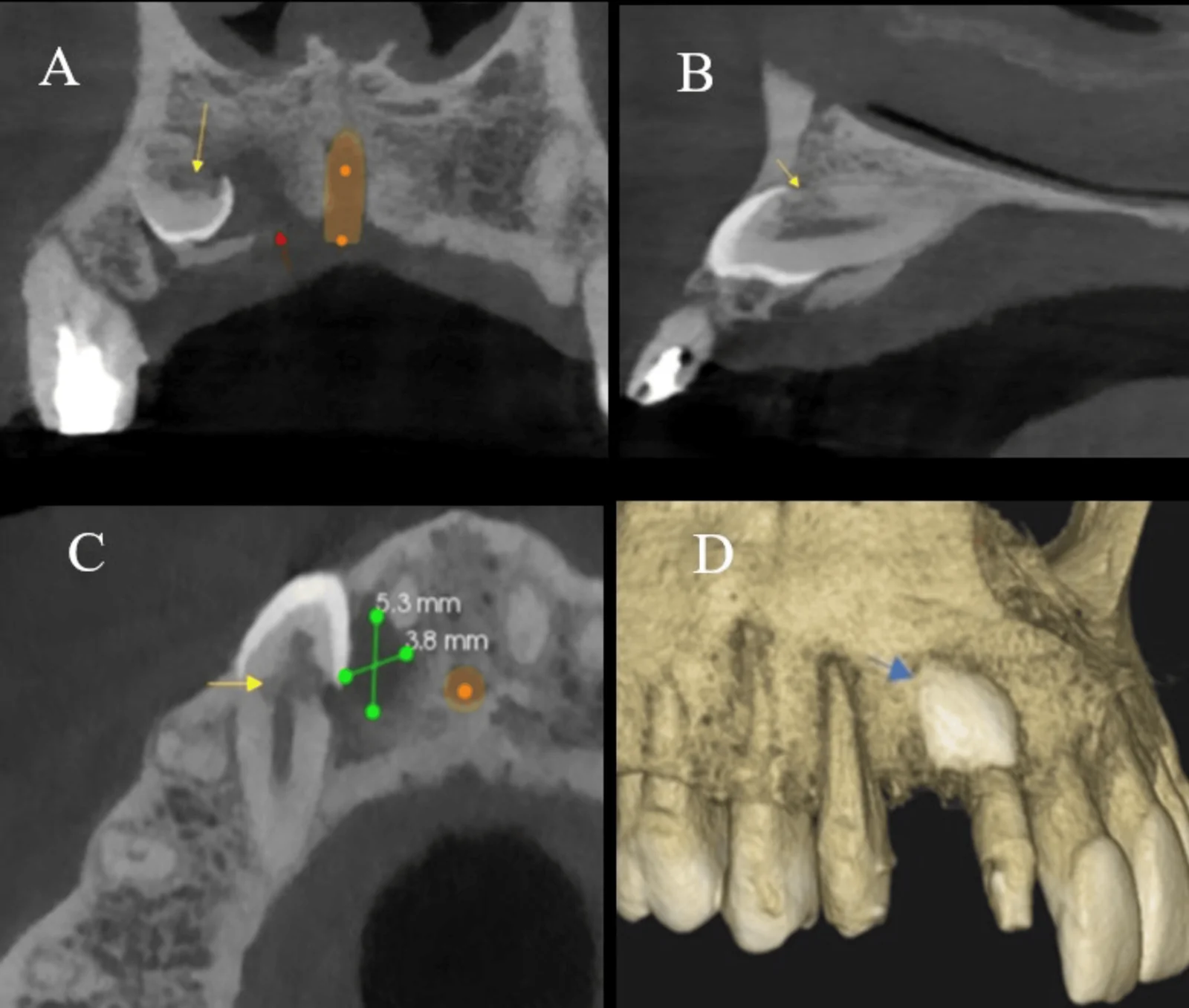
(A) CBCT sagittal section; (B) axial section; (C) coronal section; (D) 3D reconstructed CBCT image.

The differential diagnosis includes internal resorption or a pink tooth of mummery. Radiographically, internal resorption starts from the internal wall of the pulp canal, resulting in localized enlargement of pulp canal space within the affected region. It does not involve the external surface of the affected teeth. The external root resorption starts from the external surface of the root and usually has an irregular outline in the radiograph.

Clinical significance

The first case describes the unique variable radiographic appearances of external root resorption in CBCT, such as "bowtie" and "M-shaped." The second case describes an irregular "sawtooth-like" appearance. External root resorption must not be confused with cervical abrasion, which usually has a " V-shaped" radiolucent defect near CEJ and a smooth outline.

Limitations

In both cases, treatment was not done since the patient did not consent to treatment procedures as they were completely asymptomatic. In addition, the patients did not turn in for further follow-up evaluation as they hailed from a remote area.

## Discussion

ESR is a sterile, temporary, pressure-induced resorption that affects petite tooth root regions [4]. Colak et al. stated that the resorption craters analyzed by micro-CT in 26 participants were centred largely on the buccal surface and middle level of extracted contralateral upper first premolars after rapid and gradual maxillary expansion using a hyrax-type acrylic-bonded expansion device [5]. Sondeijker et al. conveyed that external apical root resorption (EARR) resulted from complicated multifactorial interactions between patients and treatment-related variables. A radiological examination is required to identify an EARR. However, there is no consensus on the type or timing of radiographs required to detect the first EARR during orthodontic therapy [6]. Patel et al. observed that external surface resorption occurs near an impacted tooth, cyst, or tumour and apically in orthodontically treated teeth. The clinical feature of teeth affected by external surface resorption was that they appeared healthy and normally responded to pulp vitality tests. The radiographic features of such teeth affected by external surface resorption include asymmetrical loss of root, blunting or flattening of the apex of the root, and an intact root canal [7]. Our case also had an asymmetrical loss of root apex.

Kapoor et al. stated that EARR was associated with several biomarkers, including dentinal proteins, enzymes, cytokines, and salivary proteins. Severe resorption exhibited greater dentin sialoprotein (DSP) and resorption protein concentrations and lower granulocyte-macrophage colony-stimulating factor (GM-CSF) levels than moderate resorption. Physiologic resorption was associated with higher levels of DSP and dentin phosphophoryn (DPP). Resorbed teeth had a greater RANKL/OPG ratio and lower levels of anti-resorptive mediators such as IL-1 receptor antagonist (RA) and IL-4 [8]. Nayyer et al. stated that photobiomodulation uses near-infrared light, which triggers mitochondrial cells to produce more adenosine triphosphate (ATP) and also modify ion channels at molecular levels to influence bone and cementum remodelling mechanisms [9].

Lin et al. stated that regenerative endodontic therapy (RET) is an effective therapy technique for external inflammatory lateral resorption (EILR) in post-traumatic tooth/pulp injuries. It results in biological healing and an improved prognosis [10].

Abbott and Lin [2] stated that the process of resorption is characterized by the resorption triad, which includes a triad of the following components: (a) breakdown of external (PDL and cementum) and internal (predentin and odontoblasts) natural barriers; (b) a viable blood supply for osteoclastic cells; (c) simulating factors such as trauma, pressure from impacted teeth, odontogenic cysts, and tumours result in pulp necrosis [11].

Parrales-Bravo et al. stated that digital periapical radiographs and CBCT are helpful in the diagnosis of external root resorption. Still, digital periapical radiographs can pose difficulty in assessing external root resorption in root canal-filled teeth with guttapercha or intracanal medicaments [11]. Lin et al. stated that resorption can occur either from infectious (bacterial infections from the root canal) or non-infectious sources, in which resorption is initiated due to damage by the immune system [12]. Bardini et al. stated that composites with bioactive endodontic cement and glass ionomer cement (GIC) were frequently used in external non-surgical treatments of external resorption and the combined approach. The treatment of external resorption depends on its accessibility and extent [13]. Baena-de la Iglesia et al. reported that CBCT possessed the highest and lowest sensitivity and specificity for diagnosing external root resorption, with 42-98% and 49.3-96.3%, respectively. The lowest and highest effective dosages of dental CBCT for external root resorption diagnosis are 34 and 1073 μSv, respectively [14].

## Conclusions

External root resorption is caused by multifactorial aetiologies such as mechanical stimuli from abnormal pressure created by impacted supernumerary teeth, orthodontic tooth movement, and chemical stimuli such as bleaching, calcium hydroxide pulpotomy, overextended root canal obturation materials such as guttapercha, silver points, and intracanal medicaments. External root resorption is often misdiagnosed as cervical abrasion or caries and is often challenging for oral physicians. A thorough radiological evaluation by CBCT is essential in identifying the exact cause of external root resorption. CBCT is a gold standard and an indispensable tool for diagnosing external root resorption.
